# A dataset for vineyard disease detection via multispectral imaging

**DOI:** 10.1016/j.dib.2025.111712

**Published:** 2025-05-27

**Authors:** Alessio Saccuti, Filippo Graziosi, Dario Lodi Rizzini

**Affiliations:** a University of Parma, Parco Area delle Scienze 181/A, 43100 Parma, Italy; b Rinova, Cesena, Italy

**Keywords:** Vineyard diseases, Flavescence Dorée, Mal d’Esca, Precision agriculture, Multispectral imaging

## Abstract

The present dataset is a collection of multispectral images designed for development of detection algorithms for grapevine diseases like Flavescence dorée (FD) and Esca (ED). Although FD severely threatens viticulture, there are few public datasets and none with multispectral data collected in the field. The collected images have been taken from a frontal perspective of vineyard plants that highlights details of leaves and trunks facilitating detailed disease analysis. The data were collected using a Micasense RedEdge-P multispectral camera, capturing six spectral bands across 172 image captures of three different grapevine varieties used in Lambrusco wines: Ancellotta, Marani, and Salamino. The dataset includes raw and processed images, calibration images for the multispectral camera, annotations detailing plant health conditions, and Python-based usage examples for researchers. Potential applications include the development of machine learning algorithms for automated disease detection, image alignment techniques, and background removal methods. The dataset is a valuable resource for advancing remote and proximal sensing in precision agriculture.

Specifications Table

**Table:** 

Subject	Computer Sciences
Specific subject area	Detection of vineyard diseases using multispectral imaging
Type of data	.dat (Micasense RedEdge-p log files), .tif (short for .tiff)/.jpg/.png (Images), .json (annotation), .ipynb (Usage examples)
Data collection	The data was collected using a Micasense RedEdge-P multispectral camera equipped with a Downwelling Light Sensor (DLS 2). The camera was mounted on a tripod and carried by hand across several vineyards. With the assistance of agronomists, the camera was positioned in front of vineyard plants affected by either Flavescence dorée, Esca, or neither. For each plant, several shots were taken while changing the horizontal point of view. Before capturing images in a specific vineyard, calibration panel images were taken while holding the camera by hand.
Data source location	The data was collected in two vineyards belonging to the agricultural cooperative Cantine Riunite located in Correggio (Reggio Emilia, Emilia-Romagna, Italy). It is managed by the RIMLab laboratory at the University of Parma, Parco Area delle Scienze 181/A, 43100 Parma, Italy.
Data accessibility	Repository name: A Dataset for Vineyard Disease Detection via Multispectral Imaging Data identification number: 10.5281/zenodo.14936376 Direct URL to data: https://zenodo.org/records/14936376
Related research article	

## 1 Value of the Data


 

• The dataset features grapevine images which is a high-value plant used for wine production. Italy and other European nations are among the world's largest wine exporters. For this reason, diseases such as Flavescence Dorée (FD) and Esca, that cause severe damage to both the plant and the grape clusters resulting in substantial losses both in terms of quantity and quality, have a significant impact on the wine industry.
• The proposed dataset aims to capture features of healthy and diseased plants that can be used for detecting these diseases. Multispectral imaging offers an alternative to chemical analysis of plants, which can be expensive and time-consuming. Indeed, FD and Esca diseases present visible features, such as leaf stains, stripes and crumpling, as well as changes in canopy color, which can be easily captured by a camera with minimal effort. Unlike the typical use of multispectral cameras with UAVs, this dataset provides a frontal view of the plants. Detecting vineyard diseases is crucial to prevent their spread and the consequent need to cut large portions of the field.
• The provided dataset can be used for training or testing algorithms for the detection of vineyard diseases. Additionally, since the data was collected from a frontal point of view, various challenges related to its direct usage can be studied. For example, algorithms for aligning the images from the six cameras of the RedEdge-P can be explored. Alternatively, methods for removing non-relevant elements, such as background objects, can also be developed.

## 2 Background

Flavescence dorée (FD) is a grapevine yellows disease affecting vineyards with severe economic consequences on viticulture and wine production. Its diagnosis is more difficult than other diseases like Esca (ED). Thus, timely monitoring and prevention are important to avoid drastic measures like quarantine and eradication of the infected grapevine. The presence of disease is guaranteed by molecular techniques such as the polymerase chain reaction (PCR), which can be performed only in a laboratory on collected samples and is quite expensive.

Sensor technology and computer vision can provide fast, non-invasive and cost-effective preliminary diagnosis of grapevine diseases suitable for monitoring large areas. The assumption is that diseases modify the physiological structure of plants and consequently the way ground incident radiation is reflected.

Some approaches perform detection using RGB images [1,2] acquired through standard optical cameras. Multispectral images provide additional channels like infrared strongly related to vegetation activity [3]. Recent works [[3], [4], [5]] present machine learning methods for grapevine disease detection in images with four or five channels. UAVs are often used for collection of birdeye-view images allowing coverage of large areas sacrificing details of plant leaves and stems.

The machine learning algorithms applied to such detection problem are strongly dependent on data availability. In spite of the increasing scientific effort in methodology, there are very few public image datasets. Tardif et al [1] published a dataset of RGB images of grapevines affected by FD, ED or healthy. The classification is performed by experts. Ryckewaert et al [6] have published a collection of hyperspectral images of grapevine leaves. While this dataset covers a wide range of symptoms, it is focused on highly detailed images of a specific part of the plant and is unsuitable for on-the-field detection.

This proposed dataset provides multispectral images of grapevine varieties Ancelotta, Marani and Salamino used in production of Lambrusco sparkling red wine. The images have been collected from fully grown vineyards nearby Reggio Emilia in the context of POR-FSER Agrarian project sponsored by Regione Emilia-Romagna as part of collective effort to monitor and prevent spread of FD and ED.

## 3 Data Description

**Fig. 1:**
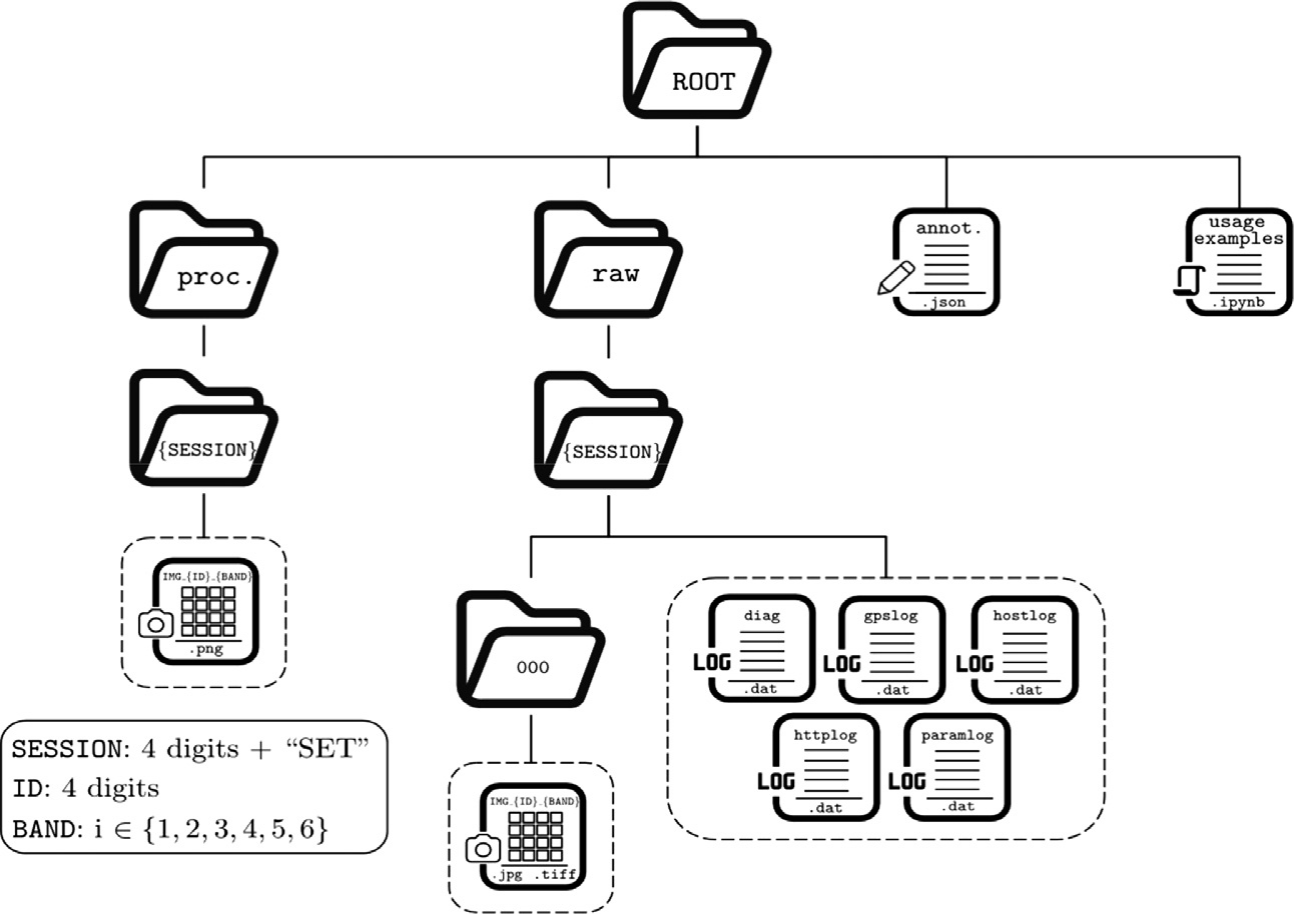
Dataset filesystem organization.

The dataset contains images of vineyards that are either healthy or affected by Flavescence dorée and Esca diseases, along with metadata, annotations, and usage examples. The data was acquired using a Micasense RedEdge-P multispectral camera. The dataset is structured as shown in Fig. 1 and can be divided into four main parts:

• Raw data folder (“raw”): it contains raw data acquired with the multispectral camera. Each subfolder, identified by a unique ID (“SESSION”), corresponds to data acquired from a specific vineyard plant. For each plant, logs were automatically recorded by the camera, and the images captured were stored in the “000” folder. Capturing an image with the Micasense RedEdge-P involves taking a picture with each of its six available cameras (typically referred as a “capture”). Therefore, for each capture, six different images were saved in the “000” folder, one for each multispectral band. These six images are stored in both '.jpg' and '.tif' formats, which are the only formats compatible with MicaSense for saving data. For both image formats, the resolution is 1456 × 1088 for the red, blue, green, near-infrared, and red-edge multispectral bands, while it is 2464 × 2056 for the panchromatic camera. In a few session folders, captures of the Micasense calibration panel are included.
• Processed data folder (“proc.”): this folder contains the output of a post-processing pipeline that takes raw data as input. The structure of the subfolders is similar to raw data folder, except that no log files are stored. In this case, the images are stored only in “.png” format, and their resolution is not predefined. The images of a processed capture have the same resolution, which varies based on the distance between the camera and the plant, as well as the presence of elements in the background.
• Annotation file (“annot.”): It contains a description of the captures contained in the “raw” directory. Essentially, it is a JSON file where each entry provides details about a vineyard plant. Specifically, each plant is annotated with a label (e.g., presence of FD or ESCA), the vineyard location, the acquisition period, the vineyard type and the number of captures taken.
• Usage examples (“usage examples”): It is a Python Jupyter notebook containing a set of code blocks that demonstrate how the data can be loaded and used. In particular, it provides examples of how data can be loaded and displayed.

The dataset contains 172 captures. In particular, the annotated plants are distributed as shown in Table 1. For each session, the number of captures is reported. In addition to the FD, ESCA, and Healthy labels, the table also includes sessions containing only calibration panel captures. Some captures are shared between different labels, e.g., 0016, which contains plants affected by both FD and ESCA, or shared between vineyard types, e.g., 0019, which contains calibration panel captures for both Ancellotta and Salamino, as the two varieties were located in the same field.

**Table 1:** Session IDs along with the number of captures contained.

Sessions (#captures)/Lambrusco type			
Label	Marani	Ancellotta	Salamino
FD	0001 (3), 0003 (4), 0005 (3), 0006 (9), 0009 (6), 0010 (5), 0011 (5), 0014 (7), 0016 (5)	0020 (6), 0021 (7)	0023 (6), 0024 (6), 0025 (5), 0026 (8), 0029 (8)
ESCA	0007 (7), 0008 (7), 0012 (5), 0015 (4), 0016 (5)	∼	0025 (5)
Healthy	0002 (3), 0004 (3), 0013 (7), 0017 (7), 0018 (9)	0022 (5)	0027 (3), 0028 (5)
Calibration panel	0000 (6)	0019 (8)	0019 (8)

## 4 Experimental Design, Materials and Methods

**Fig. 2:**
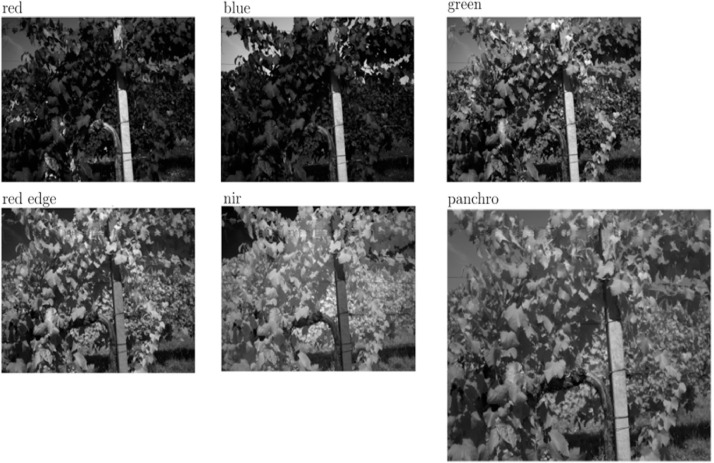
Example of a capture obtained with the multispectral camera.

The raw data in this dataset was acquired using a Micasense RedEdge-P multispectral camera. To acquire data programmatically, an experimental setup was designed. The camera was operated in combination with the HTTP API provided by Micasense [7], where HTTP requests were sent to trigger captures. An example of capture is shown in Fig. 2.

**Fig. 3:**
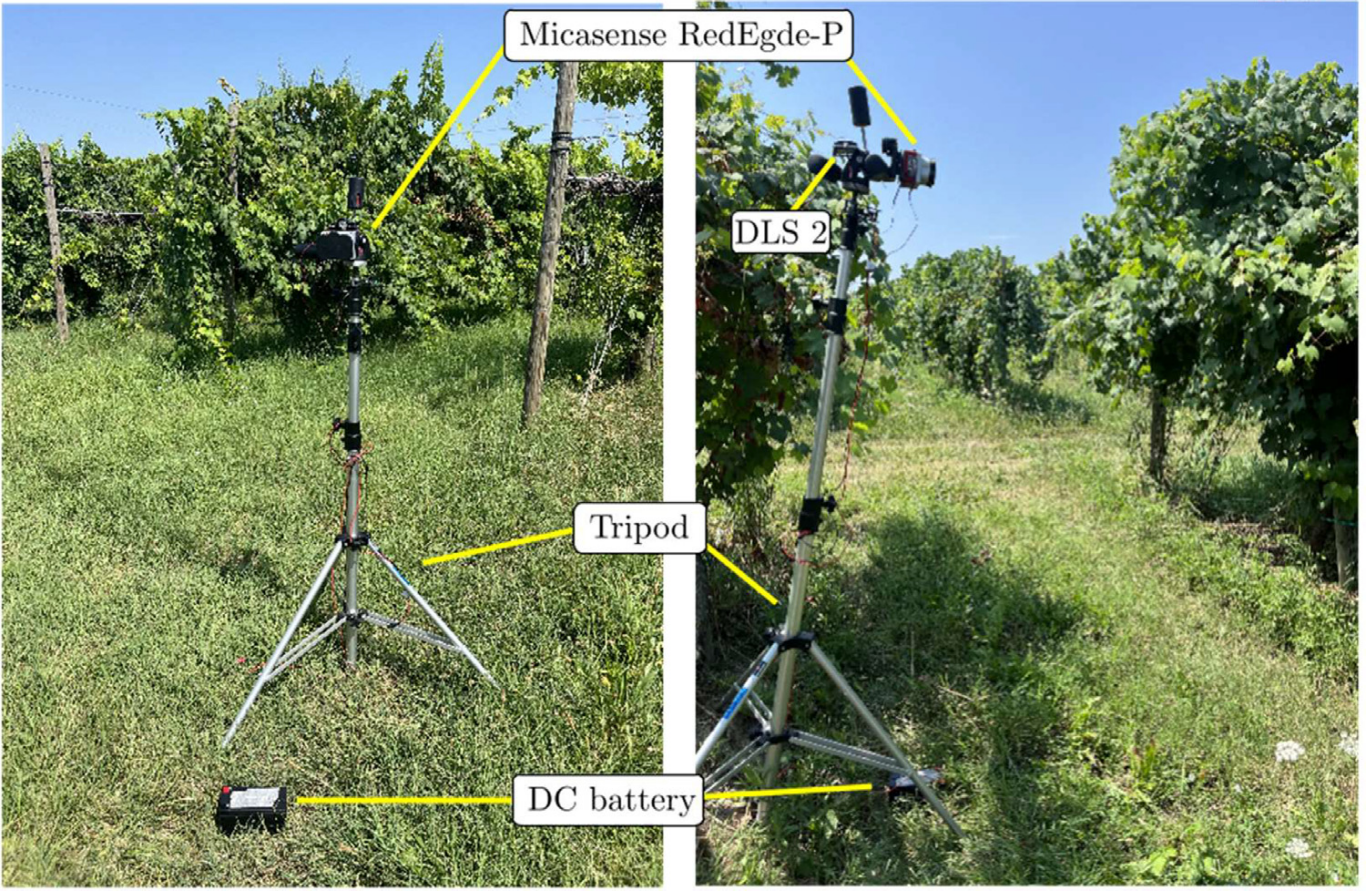
Setup used for the on-field data acquisition.

The experimental setup is shown in Fig. 3. It consists of a multispectral camera, a Downwelling Light Sensor, a tripod, and a battery. The tripod serves as a mobile platform for carrying all components except the battery. The supports used for mounting the camera, the DLS 2, and the power board were designed in Blender and 3D-printed using an Anycubic Kobra 2 NEO 3D printer with PLA+ material.

The multispectral camera is designed to create a new session folder each time it is turned on. Until it is restarted, all captures taken are stored in the most recently created session folder. To exploit this feature, we powered off and then on the camera each time we moved the tripod. In this way, we were able to automatically divide the captures by plant (or group calibration panel captures in a unique folder).

The camera was controlled using the Micasense HTTP API . The camera supports both Wi-Fi and Ethernet connections. To ease the mobility of the setup, we controlled the camera with an Apple iPad connected via Wi-Fi. To send HTTP requests, we used Python and “Carnets” app installed on the iPad. When a capture was requested, the corresponding images were saved on the SD card inserted in the camera.

The setup was moved across vineyard fields. The data acquisition was organized in the following steps:

1. First, the agronomists identified healthy or diseased plants evaluating the visual features of the plant. Plants which presented only confounding symptoms, such as sunburn or nutrient deficiencies, were labeled as healthy.
2. The setup was moved in front of the identified plant, turning off and then on the camera.
3. The iPad was used to request several captures of the plant, also changing the horizontal orientation of the camera without moving the tripod.

Before starting taking captures of a grapevine in new vineyard, captures of the calibration panel were acquired, as suggested by Micasense. To ease the acquisition of images of the panel, the camera was detached from the tripod and held by hand. During this phase, the captures were requested using the trigger button positioned on the camera.

**Fig. 4:**
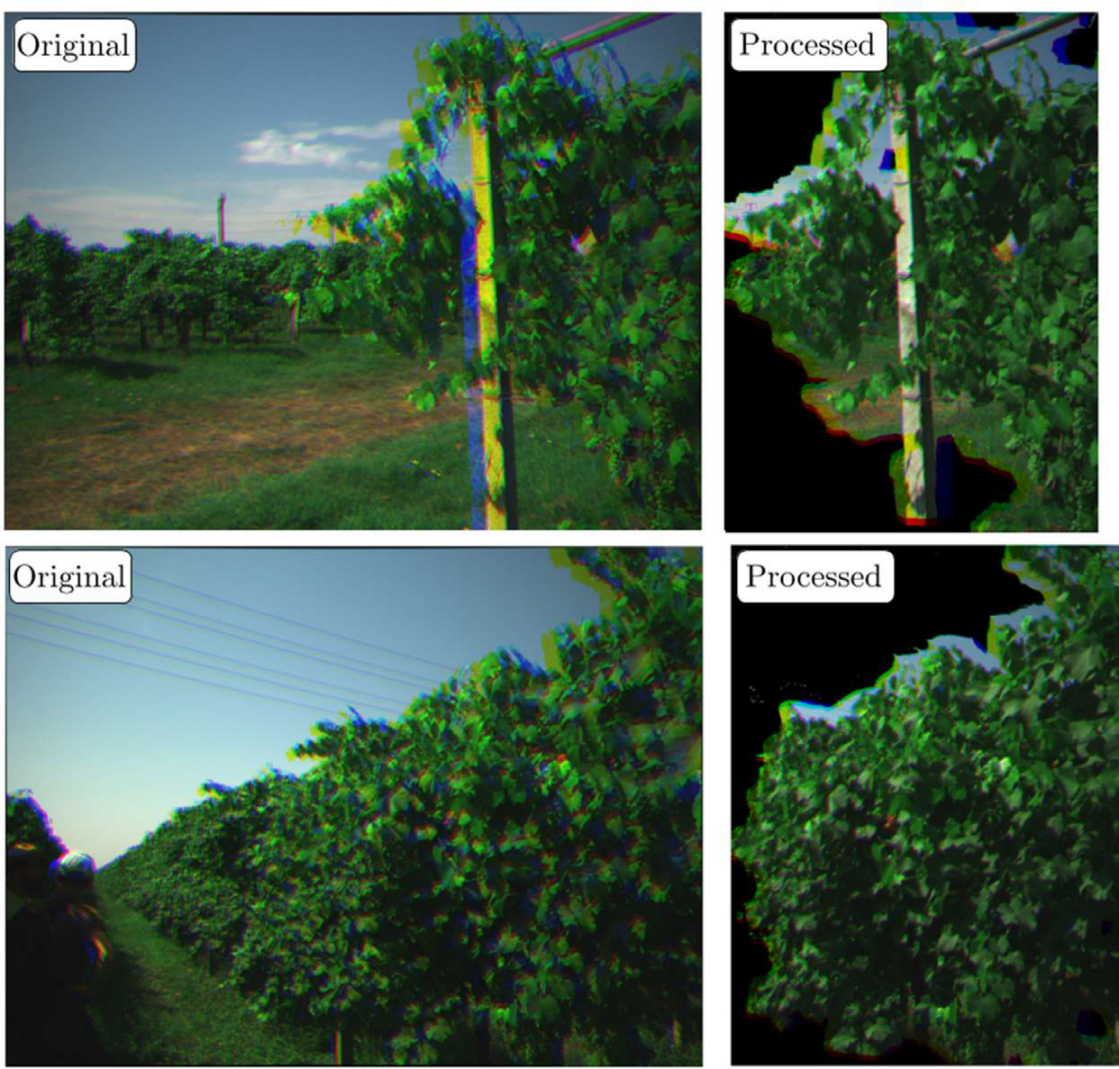
Examples of original images captured by the multispectral camera and processed images.

When the acquisition ended, the data was downloaded directly from the camera SD card. The data was then organized as mentioned above, and several attempts were made to improve its quality. In the folder of processed data, there is the result of an experimental algorithm which aims at aligning the images of a capture and removing non-relevant objects, such as the sky in the background. Examples of a raw and post-processed capture images are shown in Fig. 4, in which the red, blue, and green images are merged into RGB images for the plot. The raw images shown in the figure, which were used in post-processing, were undistorted using a Python library provided by Micasense [8].

The processed data primarily serves as an example of possible elaborations on the raw data. In literature, many works use multispectral images or simple plant images in post-processing algorithms to extract features useful for AI applications [9,10].

## Limitations

This dataset presents these limitations:

• The number of diseased and healthy plants is unbalanced.
• Due to the late acquisition period, many plants affected by FD or ESCA had developed additional symptoms (like sunburn or nutrition disease) that obscured those relevant to our study.
• The data was collected on-site, facing the typical challenges of agricultural fields. Since it was not possible to preview the camera capture, some shots may have plants that are not well centered. Additionally, due to the limited space between vineyard rows, the DLS 2 may have been obstructed in some cases, potentially leading to inaccuracies in the data.

## Ethics Statement

The authors are aware of the ethical requirements for publication in Data in Brief and confirm that the acquired data does not involve human subjects, animal experiments, or data collected from social media platforms.

## CRediT Author Statement

**Alessio Saccuti:** Conceptualization, Data Curation, Investigation, Resources, Methodology, Software, Visualization, Writing - Original Draft, Writing - Review & Editing **Filippo Graziosi:** Conceptualization, Data Curation, Investigation, Resources, Writing - Original Draft, Writing - Review & Editing. **Dario Lodi Rizzini:** Conceptualization, Data Curation, Investigation, Resources, Supervision, Project administration, Funding acquisition, Writing - Original Draft, Writing - Review & Editing.

## Data Availability

ZenodoA Dataset for Vineyard Disease Detection via Multispectral Imaging (Original data)
